# Pharmacokinetics of ß-d-N4-Hydroxycytidine, the Parent Nucleoside of Prodrug Molnupiravir, in Nonplasma Compartments of Patients With Severe Acute Respiratory Syndrome Coronavirus 2 Infection

**DOI:** 10.1093/cid/ciac199

**Published:** 2022-03-10

**Authors:** Richard FitzGerald, Laura Dickinson, Laura Else, Thomas Fletcher, Colin Hale, Alieu Amara, Lauren Walker, Sujan Dilly Penchala, Rebecca Lyon, Victoria Shaw, William Greenhalf, Katie Bullock, Lara Lavelle-Langham, Helen Reynolds, Wendy Painter, Wayne Holman, Sean Ewings, Gareth Griffiths, Saye Khoo

**Affiliations:** National Institute for Health Research Royal Liverpool & Broadgreen Clinical Research Facility, Liverpool University Hospital NHS Foundation Trust, Liverpool, United Kingdom; Department of Pharmacology & Therapeutics, University of Liverpool, Liverpool, United Kingdom; Department of Pharmacology & Therapeutics, University of Liverpool, Liverpool, United Kingdom; Department of Clinical Sciences, Liverpool School of Tropical Medicine, Liverpool, United Kingdom; National Institute for Health Research Royal Liverpool & Broadgreen Clinical Research Facility, Liverpool University Hospital NHS Foundation Trust, Liverpool, United Kingdom; Department of Pharmacology & Therapeutics, University of Liverpool, Liverpool, United Kingdom; National Institute for Health Research Royal Liverpool & Broadgreen Clinical Research Facility, Liverpool University Hospital NHS Foundation Trust, Liverpool, United Kingdom; Department of Pharmacology & Therapeutics, University of Liverpool, Liverpool, United Kingdom; National Institute for Health Research Royal Liverpool & Broadgreen Clinical Research Facility, Liverpool University Hospital NHS Foundation Trust, Liverpool, United Kingdom; Department of Pharmacology & Therapeutics, University of Liverpool, Liverpool, United Kingdom; Department of Pharmacology & Therapeutics, University of Liverpool, Liverpool, United Kingdom; Department of Pharmacology & Therapeutics, University of Liverpool, Liverpool, United Kingdom; Department of Pharmacology & Therapeutics, University of Liverpool, Liverpool, United Kingdom; Department of Pharmacology & Therapeutics, University of Liverpool, Liverpool, United Kingdom; Ridgeback Biotherapeutics, Miami, Florida, USA; Ridgeback Biotherapeutics, Miami, Florida, USA; National Institute for Health Research Southampton Clinical Trials Unit, University of Southampton, Southampton, United Kingdom; National Institute for Health Research Southampton Clinical Trials Unit, University of Southampton, Southampton, United Kingdom; National Institute for Health Research Royal Liverpool & Broadgreen Clinical Research Facility, Liverpool University Hospital NHS Foundation Trust, Liverpool, United Kingdom; Department of Pharmacology & Therapeutics, University of Liverpool, Liverpool, United Kingdom

**Keywords:** molnupiravir, pharmacokinetics, nonplasma, SARS-CoV-2, COVID-19

## Abstract

ß-d-N4-hydroxycytidine (NHC), the parent nucleoside of molnupiravir, a COVID-19 antiviral, was quantified at SARS-CoV-2 transmission sites in 12 patients enrolled in AGILE Candidate-Specific Trial-2. Saliva, nasal, and tear NHC concentrations were 3%, 21%, and 22% that of plasma. Saliva and nasal NHC were significantly correlated with plasma (*P* < .0001).

**Clinical Trials Registration**. NCT04746183.

An extended therapeutic goal of antiviral therapy is the prevention of infection in individuals who have been exposed to an infected person. Severe acute respiratory syndrome coronavirus 2 (SARS-CoV-2) infection occurs through inhalation or inoculation of virus onto upper respiratory airways and mucosal surfaces. In order to be an effective prophylactic agent, a drug must penetrate into these sites in sufficient quantities.

Molnupiravir (EIDD-2801; MK-4482), a prodrug of the ribonucleoside analogue β-d-N4-hydroxycytidine (NHC), has recently been licensed in the United Kingdom and received US Food and Drug Administration FDA (FDA) emergency use authorization (EUA) in the United States for the treatment of symptomatic coronavirus disease 2019 (COVID-19) in adults with at least 1 risk factor for developing severe disease. Following oral administration, molnupiravir is rapidly hydrolyzed by esterases to NHC, which is phosphorylated by host kinases to active intracellular metabolite EIDD-1931-5’-triphosphate (EIDD-2061) [1]. AGILE, a UK platform for early-phase trials of novel COVID-19 therapies [2], has evaluated molnupiravir within its AGILE Candidate-Specific Trial (CST)-2 seamless phase 1b/2a protocol. We previously reported phase 1b evaluation of molnupiravir across 3 dosing arms (300, 600, and 800 mg twice daily), establishing that 800 mg twice daily for 5 days was suitable for progression to phase 2 [3], which is currently recruiting.

Here, we report the pharmacokinetics of molnupiravir and NHC in saliva, nasal secretions, and tears in comparison with plasma concentrations within AGILE CST-2 1b.

## METHODS

### Study Design, Sampling, and Bioanalytical Methods

Molnupiravir pharmacokinetics were evaluated as part of a phase 1 dose-escalation study (300, 600, and 800 mg twice daily) in patients with polymerase chain reaction–confirmed SARS-CoV-2 infection within 5 days of symptom onset and presenting with mild or moderate disease. The study design has previously been described [3], and further details can be found in the Supplementary Materials.

Plasma and nonplasma (saliva, nasal secretions, and tears) samples were collected at pre-dose and 0.5, 1, 2, and 4 hours post-dose on study days 1 and 5. Plasma samples were collected as previously described [4]. Nonplasma sample collections and molnupiravir dosing conditions are described in the Supplementary Materials.

Molnupiravir and NHC concentrations were determined at the University of Liverpool Bioanalytical Facility (United Kingdom). Plasma and saliva concentrations were quantified using a validated liquid chromatography–mass spectrometry method [4]. NHC in nasal secretions and tears (swabs) were determined using an adaptation of this method, described in the Supplementary Materials. All assays were validated in accordance with FDA [5] and EMA guidelines [6].

### Pharmacokinetic Data Analyses

Given the small sample size, representation of the pharmacokinetic data was largely exploratory and descriptive. Samples below the lower limit of quantification (LLQ; <2.5 ng/mL) at pre-dose on day 1 were included as 0 ng/mL; those less than the LLQ beyond pre-dose on day 1 were included as LLQ/2 (1.25 ng/mL). NHC area under the concentration-time curve 0–4 hours (AUC_0-4_), maximum concentration (C_max_), and time to maximum concentration (T_max_) were determined using noncompartmental analysis (Phoenix 64, WinNonlin, v. 8.3, Certara, Princeton, NJ). NHC intercompartmental nonplasma:plasma ratios (R_NP:P_) were calculated on day 1 and day 5 for each individual using the plasma as reference (nonplasma AUC_0-4_/plasma AUC_0-4_). Patients without a full profile between 0 and 4 hours were excluded from AUC_0-4_ summary statistics, and those with sample(s) missing between 0 and 2 hours were excluded from C_max_ and T_max_ summary statistics.

Linear mixed-effects models were applied to evaluate the relationship between log-transformed NHC concentrations in plasma and nonplasma compartments on day 1 and day 5 (IBM SPSS Statistics v. 25.0, IBM Corporation, Armonk, NY). Concentrations below the assay LLQ were excluded.

## RESULTS

### Patients

Of the 12 participants (n = 4 per dosing arm), 9 (75%) were female, median (range) baseline age was 50 years (22–80), median (range) baseline weight was 79 kg (54–134), and median (range) baseline body mass index was 29 kg/m^2^ (21–51). Time from symptom onset to randomization and start of treatment was 5 days (3–5).

Ten of the 12 individuals (83%) completed the full treatment schedule. One patient in the 300-mg cohort took 1 of 2 tablets for the second and third dose, and a participant in the 800-mg cohort withdrew after the second dose. All pharmacokinetic data were included.

### Nonplasma Samples

Molnupiravir was detected at very low concentrations in only 31 of 106 (29%) plasma and 12 of 114 (11%) saliva samples (median [range], 5.89 [2.59–27.53 ng/mL] and 4.86 [2.63–31.44 ng/mL], respectively) and therefore not measured in swabs.

In total, 111 of 113 saliva, 112 of 112 nasal, and 96 of 106 tear concentrations were included. Sample numbers per cohort are summarized in Supplementary Table 1, and exclusions and samples less than the LLQ are outlined in the Supplementary Materials.

### NHC Nonplasma Pharmacokinetics

NHC pharmacokinetic parameters are summarized in Table 1. Geometric mean concentration-time profiles are shown in Supplementary Figure 1; additionally, individual profiles are illustrated in Supplementary Figure 2.

**Table 1.** Geometric Mean (CV%) β-d-N4-Hydroxycytidine Pharmacokinetic Parameters From Plasma, Saliva, Nasal Swabs, and Tear Strips From Severe Acute Respiratory Syndrome Coronavirus 2–Infected Patients Following Single- (Day 1) and Multiple-Dose (Day 5) Molnupiravir 300 mg, 600 mg, and 800 mg Twice Daily

	300 mg		600 mg		800 mg	
Parameter	Day 1	Day 5	Day 1	Day 5	Day 1	Day 5
Plasma						
AUC_0–4_ (ng h/mL)	3031 (45)^a^	2328^b^	5690 (22)^c^	4368 (41)	8187 (30)	7005 (21)^c^
C_max_ (ng/mL)	1488 (31)^c^	1048 (17)^a^	2440 (17)	1865 (61)	3447 (32)	3546 (13)^c^
T_max_ (h)	2.00 (1.00–2.00)^c^	1.00 (1.00–1.00)^a^	1.00 (1.00–2.00)	1.00 (1.00–2.00)	2.00 (2.00–2.00)	2.00 (2.00–2.00)^a^
Saliva						
AUC_0–4_ (ng h/mL)	65 (109)^c^	106 (93)^c^	143 (120)	106 (77)^c^	289 (52)	237 (36)^c^
C_max_ (ng/mL)	29 (113)^c^	41 (98)	73 (127)	48 (76)^c^	134 (48)	109 (27)^c^
T_max_ (h)	1.00(1.00–2.00)^c^	1.50(1.00–2.00)	2.00(1.00–2.00)	2.00(2.00–4.00)^c^	2.00(1.00–2.00)	2.00(2.00–2.00)^c^
R_NP:P_	0.03 (62)^a^	0.03^b^	0.04 (79)^c^	0.03 (51)^c^	0.04 (33)	0.03 (18)^c^
Nasal swabs						
AUC_0–4_ (ng h/mL)	1061 (38)^c^	673 (27)^c^	629 (64)	716 (67)	2164 (50)	1611 (73)^c^
C_max_ (ng/mL)	805 (70)^c^	484 (60)	365 (75)	321 (65)	1076 (43)	738 (87)^c^
T_max_ (h)	1.00 (1.00–1.00)^c^	1.00 (1.00–2.00)	1.00 (1.00–2.00)	1.00 (1.00–4.00)	1.50 (1.00–2.00)	2.00 (2.00–4.00)^c^
R_NP:P_	0.41 (73)^a^	0.23^b^	0.17 (27)^c^	0.17 (112)	0.26 (26)	0.23 (67)^c^
Tear strips						
AUC_0–4_ (ng h/mL)	1731 (44)^c^	1071 (38)^c^	1137 (96)^c^	749 (50)^a^	1934 (90)^c^	722^b^
C_max_ (ng/mL)	908 (58)^c^	674 (53)^c^	411 (100)^c^	508 (84)	985 (95)^c^	1267 (40)^c^
T_max_ (h)	0.50 (0.50–2.00)^c^	1.00 (0.50–2.00)^c^	2.00 (0.50–4.00)^c^	1.50 (1.00–2.00)^a^	1.00 (1.00–1.00)^c^	1.00 (0.50–1.00)^c^
R_NP:P_	0.77 (36)^a^	0.39^b^	0.20 (76)^c^	0.17 (47)^a^	0.26 (121)^c^	0.10^b^

n = 4 per dosing arm, unless stated otherwise. T_max_ expressed as median (range).

Abbreviations: AUC_0–4_, area under the concentration-time curve over 0 hours (pre-dose) to 4 hours post-dose; C_max_, maximum concentration; R_NP:P_, intercompartmental ratio of nonplasma to plasma (nonplasma AUC_0-4_/plasma AUC_0-4_); T_max_, time of maximum concentration.

n = 2.

n = 1.

n = 3.

NHC saliva concentrations were approximately 3% that of plasma (median [range] R_NP:P_ pooled across doses: 0.03 [0.01–0.11]; n = 16); the majority of individual ratios were between 0.01 and 0.04 (n = 12). Individual NHC R_NP:P_ for nasal secretions and tears were more variable (coefficient of variation [CV]: 60%, 70%, and 92% for saliva, nasal, and tears R_NP:P_, respectively) and, overall, approximately 6-fold higher than saliva R_NP:P_ (median [range] R_NP:P_ nasal: 0.21 [0.05–0.73]; n = 17 and tears: 0.22 [0.09–1.05]; n = 12). Geometric mean (CV%) NHC R_NP:P_ stratified by molnupiravir dose and study day are described in Table 1.

NHC concentrations in saliva and nasal secretions were significantly associated with paired plasma on day 1 and day 5 (*P* < .0001 for all analyses), whereas statistically significant relationships were not observed for paired tear and plasma NHC concentrations (day 1, *P* = .068; day 5, *P* = .344). Time post-dose was included as a repeated effect, but addition of random effects for intercept and slope did not improve the models.

## DISCUSSION

Molnupiravir, along with nirmatrelvir/ritonavir (Paxlovid), are orally administered antivirals licensed in the United Kingdom and with FDA EUA in the United States for early treatment of mild to moderate COVID-19 in adults with at least 1 risk factor for developing severe disease. Molnupiravir is currently under phase 2 evaluation within AGILE including mild to moderate COVID-19 without risk factors and in both unvaccinated and vaccinated patients. Molnupiravir is also being investigated for prophylactic use in household contacts of symptomatic COVID-19 patients (MOVe-AHEAD). Knowledge of drug accumulation within the upper airways and mucosal secretions will inform and support future research in this area.

We observed saliva NHC concentrations that were 3% that of plasma, whereas exposure in nasal secretions and tears was higher at approximately 20% that of plasma (based on pooled AUC_0-4_ ratios). Of the measured saliva, nasal, and tear samples, 6%, 50%, and 61%, respectively, were within or above the NHC 90% effective concentration (EC_90_) against SARS-CoV-2 in primary human airway epithelia cultures [7, 8] (approximately 0.5–1 µM ≈ 130–260 ng/mL), suggesting that therapeutic concentrations are potentially attained within the nasal and ocular compartments but not in saliva. However, it is important to note that without established pharmacokinetic/pharmacodynamic relationships or virological data, further studies are warranted to determine whether efficacious or prophylactic targets are obtained in nonplasma compartments.

NHC appeared to exhibit similar absorption and elimination profiles in the matrices studied, confirmed by statistically significant linear relationships between plasma NHC with that in nonplasma compartments (with the exception of tears). A strong correlation between saliva and plasma NHC concentrations implies (assuming a 1-compartment model) that salivary accumulation is dependent on the plasma concentration. Mucosal permeability and protein binding are major factors in determining salivary drug accumulation, since only unbound drug is available for diffusion into saliva [9]. NHC exists predominantly in unbound form in plasma (unbound fraction ≥0.99), and in vitro studies demonstrated that molnupiravir and NHC are not substrates for major drug transporters (eg, ABCB1, p-glycoprotein) [10, 11]. However, NHC is a substrate for human nucleoside transporters in vitro (eg, CNT1, ENT2) [12], which could modulate nonplasma concentrations of NHC in addition to other factors relating to the drug characteristics or surrounding milieu. Passage of drugs into nonplasma compartments can also be attributed to factors such as pH (eg, mouth), inflammation (eg, eye), and flow rate. For example, pharmacokinetics of drug in tears may be affected due to increased lacrimation or infection. Higher turnover or flow rate of saliva may also contribute to the lower concentrations observed. Additionally, the marked variability in nasal and tear NHC concentrations could be associated with the challenging collection procedures.

There are a number of limitations of this study. The small sample size, which is typical of early-phase studies, only allowed for a descriptive interpretation of NHC pharmacokinetics and was underpowered for statistical comparisons between matrices. We used a truncated sampling schedule between 0 and 4 hours to limit infection risk; therefore, NHC elimination over the 12-hour dosing interval could not be fully assessed in nonplasma compartments. Missing samples led to exclusions from the analysis, particularly for evaluation of R_NP:P_; contributed to data variability; and limited data interpretation. Finally, the active triphosphate metabolite, EIDD-2061, was not quantified. Despite these limitations, these data add to our understanding of NHC pharmacokinetics, principally at sites of COVID-19 infection.

To our knowledge, we are the first to describe penetration of NHC into nasal secretions and tears and, to a lesser extent, into saliva. These data support the evaluation of molnupiravir as prophylaxis for SARS-CoV-2 infection.

## Supplementary Data

Supplementary materials are available at *Clinical Infectious Diseases* online. Consisting of data provided by the authors to benefit the reader, the posted materials are not copyedited and are the sole responsibility of the authors, so questions or comments should be addressed to the corresponding author.

ciac199_suppl_Supplementary_Material

## Notes

***Acknowledgments.*** The authors thank the patients who participated in the AGILE Candidate-Specific Trial-2 molnupiravir trial, all staff involved at the National Institute for Health Research (NIHR) Royal Liverpool and Broadgreen Clinical Research Facility, and the team at the Liverpool Bioanalytical Facility who were involved in the processing and analysis of the pharmacokinetic samples, including Elizabeth Challenger and Deirdre Egan. They are grateful to the team at the Southampton Clinical Trials Unit who were involved in study coordination, including Nichola Downs, Geoffrey Saunders, Andrea Corkhill, Kerensa Thorne, Lucy Johnson, Sata Yeats, Kim Mallard, Mike Radford, and Keira Fines.

***Financial support.*** This work was funded by the University of Liverpool. The AGILE platform infrastructure is supported by the Medical Research Council (grant MR/V028391/1) and the Wellcome Trust (grant 221590/Z/20/Z). The AGILE CST-2 molnupiravir trial was supported by Ridgeback Biotherapeutics. The authors acknowledge NIHR infrastructure funding for the Liverpool Clinical Research Facility and the Southampton Clinical Trials Unit.

***Potential conflicts of interest.*** S. K. has received research funding from ViiV Healthcare, Gilead Sciences, and Merck for the Liverpool HIV Drug Interactions Programme and for clinical studies unrelated to the submitted work; funding and study material for a clinical trial awarded to their institution from GlaxoSmithKline/Vir; funding for a clinical trial awarded to their institution from UNITAID and MSD; funding support for the Liverpool COVID19 Drug Interactions Prescribing Tool, awarded to their institution from AbbVie, MSD, Novartis, and Swedish Orphan Biovitrum AB (SOBI) all unrelated to the submitted work; consulting fees from ViiV; and speakers honoraria from ViiV, Gilead, and AbbVie. G. G. has received funding from Jannsen-Cilag, Novartis, Astex, Roche, Heartflow, Bristol Myers Squibb, and BioNtech; grants and personal fees from AstraZeneca; and personal fees from Celldex, unrelated to the submitted work and reports that the academic Clinical Trial Unit (no personal payment) receives Ridgeback Therapeutics funding for the running of the academically led and sponsored AGILE trial platform including molnupiravir. W. G. has received funding from the Wellcome Trust and reports support for the current work from GlaxoSmithKline and UK Research & Innovation (UKRI) to support AGILE platform clinical investigator Saye Khoo. W. H. is a cofounder, owner, and advisor of/to Ridgeback Biotherapeutics and reports being a shareholder for Merck; is listed as an inventor on a patent application relating to molnupiravir; and is co-chair of the Ridgeback Biotherapeutics–Merck Molnupiravir Joint Steering Committee. W. P. is employed by Ridgeback Biotherapeutics and reports royalties or licenses from George Painter, inventor of molnupiravir (family member) and prior consulting work (before this study) from Emory University (Emory University Institute for Drug Development [EIDD], Drug Innovation Ventures at Emory [DRIVE]). W. H. and W. P. P. are, along with others, presently named as coinventors of 2 pending provisional patent applications, “Treatment of Viruses with Antiviral Nucleosides,” submitted on behalf of Ridgeback Biopharmaceuticals, Emory University, and Merck Sharp & Dohme Corp. T. F. reports grants or contracts from Ridgeback, MRC, Wellcome Trust, and all outside of the submitted work. All other authors report no potential conflicts. All authors have submitted the ICMJE Form for Disclosure of Potential Conflicts of Interest. Conflicts that the editors consider relevant to the content of the manuscript have been disclosed
